# Minimum effective dose of clemastine in a mouse model of preterm white matter injury

**DOI:** 10.1038/s41390-024-03326-w

**Published:** 2024-06-28

**Authors:** Elizabeth P. Odell, Nora Jabassini, Björn Schniedewind, Sarah E. Pease-Raissi, Adam Frymoyer, Uwe Christians, Ari J. Green, Jonah R. Chan, Bridget E. L. Ostrem

**Affiliations:** 1 Weill Institute for Neurosciences, Department of Neurology, University of California, San Francisco, San Francisco, CA USA; 2 iC42 Clinical Research and Development, Department of Anesthesiology, University of Colorado Anschutz Medical Campus, Aurora, CO USA; 3 Department of Pediatrics, Stanford University, Palo Alto, CA USA; 4 Department of Ophthalmology, University of California, San Francisco, San Francisco, CA USA

## Abstract

**Background:**

Preterm white matter injury (PWMI) is the most common cause of brain injury in premature neonates. PWMI involves a differentiation arrest of oligodendrocytes, the myelinating cells of the central nervous system. Clemastine was previously shown to induce oligodendrocyte differentiation and myelination in mouse models of PWMI at a dose of 10 mg/kg/day. The minimum effective dose (MED) of clemastine is unknown. Identification of the MED is essential for maximizing safety and efficacy in neonatal clinical trials. We hypothesized that the MED in neonatal mice is lower than 10 mg/kg/day.

**Methods:**

Mouse pups were exposed to normoxia or hypoxia (10% FiO2) from postnatal day 3 (P3) through P10. Vehicle or clemastine at one of four doses (0.5, 2, 7.5 or 10 mg/kg/day) was given to hypoxia-exposed pups. Myelination was assessed at age P14 and 10 weeks to determine the MED. Clemastine pharmacokinetics were evaluated at steady-state on day 8 of treatment.

**Results:**

Clemastine rescued hypoxia-induced hypomyelination with a MED of 7.5 mg/kg/day. Pharmacokinetic analysis of the MED revealed C_max_ 44.0 ng/mL, t_1/2_ 4.6 h, and AUC_24_ 280.1 ng*hr/mL.

**Conclusions:**

Based on these results, myelination-promoting exposures should be achievable with oral doses of clemastine in neonates with PWMI.

**Impact:**

- Preterm white matter injury (PWMI) is the most common cause of brain injury and cerebral palsy in premature neonates.
- Clemastine, an FDA-approved antihistamine, was recently identified to strongly promote myelination in a mouse model of PWMI and is a possible treatment.
- The minimum effective dose in neonatal rodents is unknown and is critical for guiding dose selection and balancing efficacy with toxicity in future clinical trials.
- We identified the minimum effective dose of clemastine and the associated pharmacokinetics in a murine chronic hypoxia model of PWMI, paving the way for a future clinical trial in human neonates.

## Introduction

Preterm white matter injury (PWMI) affects approximately 20,000 neonates in the United States annually and can result in lifelong motor and cognitive disability.^1–3^ There are no specific treatments available; symptom-directed supportive care is the mainstay of management.^4^ Oligodendrocytes (OLs) and their precursors (oligodendrocyte precursor cells, OPCs) comprise the major cell types implicated in PWMI, which involves an arrest of differentiation of OPCs and a reduction in mature OLs and myelin formation.^5–14^ The OL lineage is therefore an ideal target for therapeutics aimed at promoting recovery in the setting of PWMI.

A previous high-throughput screen using a novel micropillar array-based assay identified several FDA-approved compounds, including the antihistamine clemastine, that promote differentiation of OPCs into mature, myelinating OLs.^15^ Clemastine at doses of 10 mg/kg/day was further demonstrated to enhance myelination and functional recovery in several animal models of white matter injury, including PWMI.^16–22^ However, the minimum effective dose (MED) that promotes neonatal brain repair in mice is unknown. Identification of the MED is essential to guide therapeutic development and limit the chance of adverse events in future clinical trials. A pharmacokinetic (PK) understanding of the MED in mice can further aid in determining the optimal dosing scheme and target exposure in humans.

Here, we use a chronic hypoxia model in neonatal mice that recapitulates major features of PWMI, including diffuse hypomyelination, OL maturation delay and persistent motor and cognitive deficits.^21–23^ PWMI is thought to result from cumulative brain insults, including hypoxia, in premature babies during the neonatal intensive care nursery hospitalization (typically from birth through approximately 40 weeks postmenstrual age).^24^ We therefore subjected neonatal mice to chronic hypoxia from postnatal day 3 (P3) through P10, a time period where cortical development and regional myelination are analogous to approximately 23 through 40 weeks postmenstrual age in humans.^25–27^ Using the murine chronic hypoxia model, we determine the MED of clemastine, which we defined as the lowest dose that promoted myelination at P14 above vehicle control levels by all measures tested. We demonstrate durability of the effect in young adult (10-week-old) mice, and characterize the pharmacokinetics of the MED, paving the way for a future clinical trial of clemastine in neonates with PWMI.

## Methods

### Chronic hypoxia and clemastine treatment

All animal studies were approved by the University of California, San Francisco, Institutional Animal Care and Use Committee. Male and female C57BL/J mice were used in equal proportions for all experiments except where indicated otherwise. Mice were housed in temperature- and humidity-controlled environments on a 12 h/12 h light/dark cycle with free access to standard chow and water. For chronic hypoxia experiments, mouse pups with their lactating mothers were subjected to chronic sublethal hypoxia (10% fraction of inspired oxygen [FiO_2_]) from P3 through P10. Mouse pups were treated daily from P3 through P10 with vehicle (saline) or clemastine fumarate (Selleckchem) by oral gavage. Doses of clemastine (weighed and dosed based on the clemastine fumarate salt) were: 0.5, 2, 7.5, or 10 mg per kg of body weight. On P10, mice were returned to normoxic (21% FiO_2_) conditions. At P14 and 10 weeks of age, OL differentiation and myelination were compared in hypoxic mice treated with vehicle or clemastine and in mice exposed to normoxia from P0 through P14.

### Immunohistochemistry

P14 and 10-week-old mice were euthanized and perfused transcardially with ice-cold Phosphate Buffered Saline (PBS) followed by ice-cold 4% paraformaldehyde (Electron Microscopy Sciences) diluted in water. Brains and optic nerves were removed and stored overnight in 4% paraformaldehyde at 4 °C. Tissues were then placed in 30% sucrose in PBS at 4 °C for 1–2 days followed by freezing in O.C.T. compound (Tissue-Tek) and generation of 30 um sections on a microtome (HM 450 Sliding Microtome, Epredia™, Richard-Allan Scientific) or cryostat (Leica CM 1850). Sections were blocked and permeabilized for 2 h at room temperature in blocking solution (PBS with 0.1% Triton X-100, and 10% donkey or goat serum), and subsequently incubated overnight at 4 °C in blocking solution with primary antibody added. After washing, secondary antibody incubation was performed for 2 h at room temperature in 10% donkey or 10% goat serum in PBS with secondary antibody added. Primary antibodies used were: rat anti-MBP (1:500, MCA409S, Serotec), mouse anti-Olig2 (1:200, EMD Millipore, MABN50), rabbit anti-Cleaved Caspase-3 (CC3, 1:300, Cell Signaling 9661 S), rabbit anti-SOX10 (1:500, EMD Millipore AB 5727), mouse anti-CC1 (1:300 Calbiochem OP80-100uG), rabbit anti-PDGFRα (1:500, Source: W.B. Stallcup), rabbit anti-Caspr (1:500, Abcam AB34151), chicken pan anti-Neurofascin (1:500, R&D Systems, AF3235), and rabbit anti-Neurofilament heavy subunit (1:500, Abcam AB8135). Secondary antibodies were: donkey or goat AlexaFluor 488 (1:1,000), 594 (1:1000), or 647 (1:500)-conjugated IgG. Sections were counterstained with DAPI (Themofisher/Invitrogen D1306). Fluorescent images were obtained using a Zeiss Imager M2 (1024994428) microscope or a Zeiss LSM700 inverted confocal microscope. MBP and neurofilament fluorescence signal intensities were measured using Imaris (v9.3.1) in a fixed area of striatum or cortex in coronal brain sections, or in equivalent areas of longitudinally sectioned optic nerves, and normalized within each section to the area of lowest fluorescence. Cell counts were performed using Imaris (v9.3.1) software, Count Spots function, manually adjusted to remove incorrect spots (for example double counting of single cells or counting of fluorescent debris). All fluorescence intensity quantifications and cell counting were performed by an investigator blinded to the experimental condition.

### Electron microscopy

P14 mice were euthanized and perfused transcardially with ice-cold 0.1 M sodium cacodylate buffer (0.1 M sodium cacodylate trihydrate [Electron Microscopy Sciences, 12310], 5 mM calcium chloride dihydrate [Sigma, 223506], pH adjusted to 7.3–7.4), followed by ice-cold EM fixative (1.25% glutaraldehyde [Electron Microscopy Sciences, 16220], 2% paraformaldehyde [Electron Microscopy Sciences, 19210], 0.1 M sodium cacodylate buffer). Brains and optic nerves were removed and stored for 8 days in EM fixative at 4 °C, and subsequently placed in 30% sucrose in PBS at 4 °C for 1–2 days. Samples were mounted in O.C.T. compound (Tissue-Tek) and 500 um sections were generated on a microtome (HM 450 Sliding Microtome, Epredia™, Richard-Allan Scientific). For analysis of the corpus callosum, the section at approximately +1.1 mm anterior to bregma (joining of corpus callosum) was placed in PBS and under a dissecting microscope, the middle 1/3 of the corpus callosum was dissected using a razor blade. Samples were stained with osmium tetroxide, dehydrated in ethanol and embedded in TAAB resin. Brain sections were cut perpendicular to the angle of the fibers of the corpus callosum and optic nerves were cut perpendicular to the nerve, at 1-mm intervals. Axons were examined using electron microscopy, and g-ratios were calculated as the diameter of the axon divided by the diameter of the axon and the surrounding myelin sheath. We could not reliably identify unmyelinated axons, particularly in vehicle-treated animals, which would have confounded comparisons of unmyelinated axon counts and diameters. Thus, all quantifications were performed on myelinated axons only. Measurements were performed using ImageJ (v1.54i) by an investigator blinded to the experimental condition.

### Plasma sample collection and mass spectrometry

Male and female C57BL/J mice were dosed by oral gavage with 7.5 mg/kg/day clemastine daily from P3 to P10. Blood was sampled before (time=0) the 8^th^ dose and at 0.5, 1, 1.5, 2, 3, 4, 6, 9, 12, and 24 h after the 8th dose. Terminal blood collections were performed by cardiac puncture after deeply anesthetizing the animal. At least 3 animals were sampled per timepoint and at least one animal per sex was included for every time point. Blood was collected into 1 ml syringes using 25ga needles coated with 0.5 M EDTA (ThermoFisher, #15575020), and immediately gently transferred into K2EDTA tubes (Sarstedt #41.1395.105) followed by centrifugation at 6700 rcf for 5 min at 4 °C. Plasma was transferred into cryotubes (Nalgene™ Cryogenic Tube, #5012-0020) and stored at −80 °C until sample analysis.

Clemastine was quantified in mouse EDTA plasma using high-performance chromatography- tandem mass spectrometry (LC-MS/MS) at iC42 Clinical Research and Development (University of Colorado, Aurora, CO). The assay followed the principles described by ref. ^28^. Clemastine reference material was from Toronto Research Chemicals (North York, ON, Canada) and the internal standard diphenhydramine-D_3_ from Sigma Aldrich (St. Louis, MO). Isotope-labeled clemastine as internal standard was commercially not available at the time of the study. Two hundred (200) µL of a protein precipitation solution (0.2 M ZnSO_4_ 30% water/ 70% methanol v/v) containing the internal standard (1.0 ng/mL diphenhydramine-D_3_) was added to 50 µL of study samples, quality control samples, calibrators and zero samples. Samples were vortexed for 2.5 min, centrifuged at 4 °C and 16,000 g for 10 min. The supernatants were transferred into 2 mL glass HPLC injection vials. The samples were then further extracted online and analyzed using a 2D-LC-MS/MS system composed of Agilent 1100 HPLC components (Agilent Technologies, Santa Clara, CA) and a Sciex API 5000 MS/MS detector (Sciex, Concord, ON, Canada) connected via a turbo flow electrospray source run in the positive ionization mode (4500 V, 550 °C source temperature).

Ten (10) µL of the samples were injected onto the extraction column (Zorbax XDB C8, 4.6 · 50 mm, Agilent Technologies). The mobile phase was 80% 0.1% formic acid in HPLC grade water (mobile phase A) and 20% methanol containing 0.1% formic acid (mobile phase B). Samples were cleaned with a solvent flow of 3 mL/min and the temperature for the extraction column was set to room temperature. After 0.7 min, the switching valve was activated and the analytes were eluted in the backflush mode from the extraction column onto a 4.6 · 150 mm analytical column filled with C8 material of, 5 μm particle size (Zorbax XDB C8, Agilent Technologies). The analytes were eluted using a gradient starting with 50% mobile phase B that increased to 98% within 2.3 min and was held for 1.0 min. The system was then re-equilibrated to starting conditions at 50% B for 0.8 min. The flow rate was 1.0 mL/min and the analytical column was kept at 60 °C. The MS/MS was run in the multiple reaction mode (MRM) and the following ion transitions were monitored: clemastine *m/z* = 346.2 [M(^37^Cl) + H]^+^ → 217.0 (quantifier), *m/z* = 344.2 [M(^35^Cl) + H]^+^ → 215.0 (qualifier) and diphenhydramine-D_3_ (internal standard) *m/z* = 259.0 [M + H]^+^ → 167.3. Declustering potentials were set to 51 V and collision energies were set to 23 V for clemastine quantifier and qualifier transitions. For the internal standard diphenhydramine-D_3_ the declustering potential was 56 V and the collision energy 19 V.

Clemastine concentrations were quantified using the calibration curves that were constructed by plotting nominal concentration *versus* analyte area to internal standard area ratios (response) using a quadratic fit and 1/x weight. All calculations were carried out using the Sciex Analyst Software (version 1.7.3). The quantification range for clemastine was 0.0025 (lower limit of quantification) – 20.0 ng/mL and study sample were diluted 1:50 and 1:250 as necessary for the detector response to fall within the calibration range as necessary. All results reported were from runs that met the following acceptance criteria: >75% of the calibrators had to be within ±15% of the nominal values (except at the lower limit of quantification: ±20%) and >2/3 of the quality controls had to be within ±15% of the nominal values. The imprecision of the results was better than 15%. Significant carry-over and matrix effects were excluded and dilution integrity was established.

### Pharmacokinetic analysis

Non-compartmental PK analysis was conducted using the geometric mean of each timepoint. Steady-state conditions were assumed after 7 days of dosing. The maximum concentration (C_max_), time of the maximum concentration (T_max_), and the concentration at 24 h post dose (C_24h_) were taken directly from the observed data. Area under the curve during the 24 h dosing interval (AUC_24_) was calculated using the trapezoidal method. Oral Clearance (CL/F) was then calculated as Dose/AUC_24_. The terminal elimination rate constant (k_e_) was calculated using linear regression of log transformed concentrations over the terminal log-linear decline phase and from this the terminal elimination half-life calculated (t_1/2_ = 0.693/k_e_).

### Statistical analysis

Statistical significance between groups was determined with GraphPad Prism 5 software. Initial assessments of myelination (MBP intensity) and the OL lineage (CC1+ and SOX10+ cells) split by sex were performed using two-way analysis of variance (ANOVA) tests followed by post hoc Dunnett’s tests for pair-wise comparisons between the hypoxia plus vehicle group and all other treatment groups. All data sets used for two-way ANOVAs passed Shapiro-Wilk tests for normality of the residuals and Spearman’s tests for heteroscedasticity (to assess for equal variance) except for the data used for analysis of CC1+ cell density (Supplementary Table 1). The data set used for CC1+ cell density analysis was log transformed, and subsequently passed both tests for normality and equal variance; a two-way ANOVA was then performed on the log transformed data. The data sets for comparisons of immunohistochemistry and electron microscopy quantifications not split by sex were tested for normality using a Shapiro–Wilk test and for equal variance using a Brown–Forsythe test (Supplementary Table 1). Normally distributed datasets with equal variance were compared using one-way ANOVA followed by Tukey’s or Dunnett’s post hoc tests (depending on the number of comparisons made) for pair-wise comparisons. Datasets that were not normally distributed were compared using a Kruskal–Wallis test with post hoc Dunn’s tests. Datasets that were normally distributed and had unequal variance were compared using a Brown–Forsythe and Welch one-way ANOVA followed by Dunnett post hoc tests. Pairwise comparisons were performed between the hypoxia plus vehicle group and all other groups for experiments that tested all 6 treatment conditions (hypoxia plus vehicle, hypoxia plus clemastine at 0.5, 2, 7.5 or 10 mg/kg/day, and normoxia). Pairwise comparisons were performed between all groups for experiments that tested only the hypoxia plus vehicle, hypoxia plus clemastine MED, and normoxia conditions. We made the assumption of independence. When possible, we assigned different treatments to mice within the same litter (after an identifying toe clip at P3) to control for batch/litter effects. A probability of *p* < 0.05, adjusted for multiple comparisons, was considered significant for all statistical comparisons.

## Results

### Identification of minimum effective dose of clemastine

**Fig. 1:**
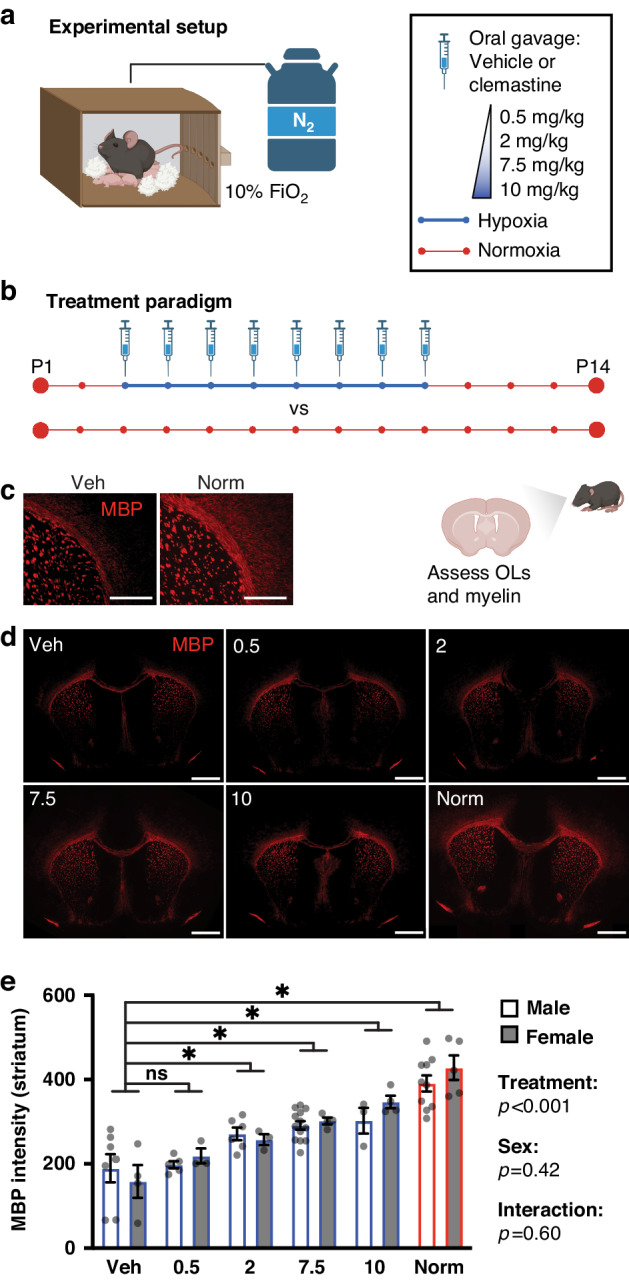
Clemastine rescues hypoxia-induced hypomyelination. **a** Schematic of hypoxia chamber, in which mice are exposed to 10% FiO_2_ via displacement of oxygen with nitrogen (N_2_) gas. Mice were placed in the chamber in their home cages and given free access to bedding, food and water. **b** Treatment paradigm. Mice were exposed to normoxia (21% FiO_2_) throughout the first 2 weeks of life, or to 10% FiO_2_ from postnatal day 3 (P3) through P10 while undergoing daily treatment with vehicle (saline) or clemastine at 0.5, 2, 7.5, or 10 mg/kg/day. At P14, oligodendrocyte density and myelination were assessed in hypoxic mice treated with vehicle or clemastine and in mice reared in normoxic conditions. **c** Representative control (vehicle plus hypoxia, and normoxia) images of corpus callosum and striatum stained for myelin basic protein (MBP) at P14. Magnification: 40X; scale bars: 200 um. **d** Representative images of coronal brain sections stained for MBP at P14. Includes lower magnification images of the same sections depicted in (**c**). Magnification: 10X; scale bars: 500 um. **e** Bar graph of mean myelin density (MBP fluorescence intensity) in the striatum by sex and treatment group. There was a significant effect of treatment, but not sex or interaction, in a two-way ANOVA of treatment group and sex (predictors) with outcome of MBP intensity. Results of pairwise comparisons between MBP intensity of the vehicle group and other treatment groups are shown above the bar graph. *p < 0.05, two-way ANOVA followed by post hoc Dunnett’s tests; ns, not significant. Error bars represent standard error of the mean (S.E.M.). For all graphs and images, hypoxia-exposed animals were: Veh (vehicle-treated), 0.5 (clemastine 0.5 mg/kg/day), 2 (clemastine 2 mg/kg/day), 7.5 (clemastine 7.5 mg/kg/day), 10 (clemastine 10 mg/kg/day). Norm, normoxia-exposed animals. Diagrams were created with BioRender.com.

To determine the lowest oral dose of clemastine that provides the full pro-myelination and OL differentiation effect of the medication, we used a murine chronic hypoxia model. Mouse pups and lactating dams were subjected to chronic sublethal hypoxia (10% fraction of inspired oxygen [FiO_2_]) from postnatal day 3 (P3) through P10 (Fig. 1a and Supplementary Table 1). This exposure leads to widespread OL maturation delay and hypomyelination, mimicking histopathological findings in human neonates.^7,8,21,29,30^ Hypoxia-exposed mice were treated once daily by oral gavage with either vehicle or clemastine from P3 through P10 and returned to normoxic (21% FiO_2_ conditions) on P10 (Fig. 1b). The highest clemastine dose used was 10 mg/kg/day, which has previously been reported to rescue hypoxia-induced hypomyelination in the murine chronic hypoxia model,^21^ and to induce (re)myelination in models of multiple sclerosis,^16^ psychiatric disorders,^17,18^ aging^31^ and neurodegenerative disorders.^32,33^ We tested a 20-fold lower dose (0.5 mg/kg/day) and two intermediate doses, 2 and 7.5 mg/kg/day. Analysis of myelination and OL differentiation was performed at P14, an age where myelination is considered approximately equivalent to a 1-year-old human infant.^27^

Myelin protein expression after hypoxia was quantified as myelin basic protein (MBP) fluorescence signal intensity in the striatum (Fig. 1c–e and Supplementary Table 2). We performed a two-way ANOVA to analyze the effect of treatment group (hypoxia plus vehicle, hypoxia plus clemastine at 0.5, 2, 7.5 or 10 mg/kg/day, and normoxia) and sex on myelination, given reported sex differences in the incidence and severity of PWMI in humans^34^ and rodents.^35^ Simple main effects analyses showed that treatment group had a statistically significant effect on MBP intensity (*p* < 0.001), while sex did not (*p* = 0.42). Post hoc Dunnett’s tests between the hypoxia plus vehicle group and all other treatment groups revealed that MBP intensity was significantly higher in the 2 (*p* = 0.003), 7.5 (*p* < 0.001) and 10 (*p* < 0.001) mg/kg hypoxia groups and the normoxia group (*p* < 0.001) as compared to the hypoxia-exposed, vehicle-treated group (hereafter called the “vehicle” group). There was not a statistically significant interaction between the effects of treatment group and sex on MBP intensity (*p* = 0.60). We also quantified corpus callosum area at P14 on coronal sections stained for MBP (Supplementary Fig. 1a). Corpus callosum area was significantly increased in hypoxia plus 7.5 and 10 mg/kg clemastine groups and the normoxia group as compared to the vehicle group (*p* < 0.05, one-way ANOVA followed by post hoc Dunnett’s tests).

**Fig. 2:**
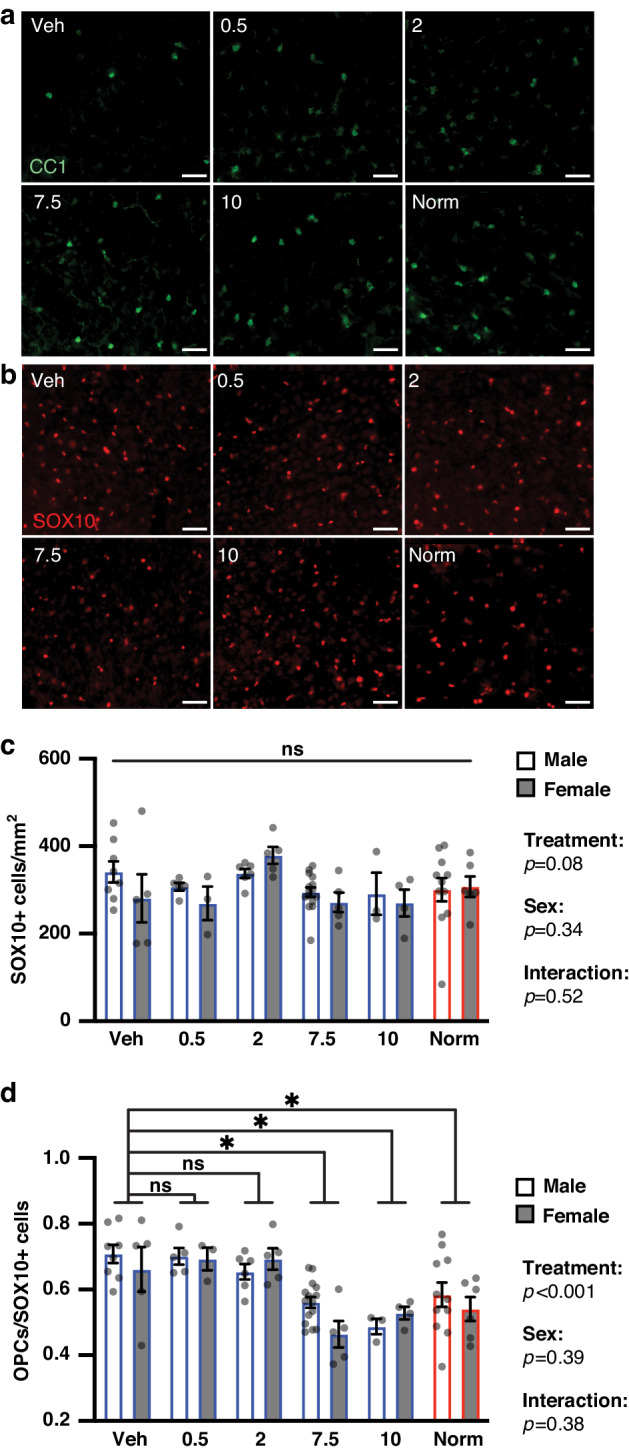
Clemastine rescues hypoxia-induced deficits in oligodendrocyte differentiation. **a** Representative images of staining for mature (CC1+) oligodendrocytes in the cortex of P14 mice from each treatment condition. Magnification: 20X; scale bars, 50 um. **b** Representative images of staining for oligodendrocyte lineage (SOX10+) cells in the cortex of P14 mice from each treatment condition. Magnification: 20X; scale bars, 50 um. **c** Bar graph of mean SOX10+ cell density in cortex by sex and treatment group. There was not a significant effect of treatment group, sex or interaction in a two-way ANOVA of treatment group and sex (predictors) with outcome of SOX10+ cell density; ns, not significant. Error bars represent S.E.M. **d** Bar graph of mean proportion of oligodendrocyte precursor cells (OPCs, defined as CC1-/SOX10+ cells) out of all oligodendrocyte lineage cells in cortex by sex and treatment group. There was a significant effect of treatment group, but not sex or interaction, in a two-way ANOVA of treatment group and sex (predictors) with outcome of CC1-/SOX10+ out of SOX10+ cells. Results of pairwise comparisons between mean OPCs out of SOX10+ cells of the vehicle group and other treatment groups are shown above the bar graph. *p < 0.05, two-way ANOVA followed by post hoc Dunnett’s tests; ns, not significant. Error bars represent S.E.M. For all graphs and images, hypoxia-exposed animals were: Veh (vehicle-treated), 0.5 (clemastine 0.5 mg/kg/day), 2 (clemastine 2 mg/kg/day), 7.5 (clemastine 7.5 mg/kg/day), 10 (clemastine 10 mg/kg/day). Norm, normoxia-exposed animals.

We also tested whether clemastine treatment at doses lower than 10 mg/kg rescued hypoxia-induced OL maturation deficits at P14. Mature (CC1+) OLs and total OL lineage (SOX10+) cells were quantified in hypoxia-exposed animals treated with vehicle or clemastine at 0.5, 2, 7.5 or 10 mg/kg/day, and in normoxia-exposed animals (Fig. 2a, b). We performed two-way ANOVAs to analyze the effect of treatment group and sex on mature OL density, total OL lineage cell density, and the proportion of OPCs (defined as CC1-/SOX10+ cells) out of total OL lineage cells (Fig. 2c, d, Supplementary Fig. 1b, and Supplementary Tables 3–5). Simple main effects analyses showed that treatment group had a statistically significant effect on CC1+ cell density (*p* < 0.001) and OPCs/SOX10+ cells (*p* < 0.001), but not SOX10+ cell density (*p* = 0.08). Pairwise analysis by post hoc Dunnett’s tests revealed that CC1+ cell density was significantly higher in the 7.5 (*p* = 0.001) and 10 (*p* = 0.012) mg/kg clemastine-treated hypoxia groups and the normoxia group (*p* = 0.011) as compared to the vehicle group. The proportion of OPCs out of total OL lineage cells was also significantly lower in the 7.5 (*p* < 0.001) and 10 (*p* < 0.001) mg/kg clemastine-treated hypoxia groups and the normoxia group (*p* = 0.002) as compared to the vehicle group (post hoc Dunnett’s tests). There was not a significant effect of sex on CC1+ cell density (*p* = 0.53), SOX10+ cell density (*p* = 0.34), or proportion of OPCs out of SOX10+ cells (*p* = 0.39). There was also not a statistically significant interaction between the effects of treatment group and sex on CC1+ cell density (*p* = 0.42), SOX10+ cell density (*p* = 0.52), or proportion of OPCs/SOX10+ cells (*p* = 0.38). Given the absence of a significant effect of sex on myelination or OL differentiation, we did not split mice by sex in further analyses. However, these negative results do not rule out the possibility that sex impacts the brain response to hypoxia or myelination-promoting treatments. In summary, clemastine treatment at 7.5 mg/kg/day and above rescued hypoxia-induced myelination deficits and OL differentiation deficits in neonatal mice. Based on our dose-response analysis, we established 7.5 mg/kg/day as the MED of clemastine in our murine chronic hypoxia model of PWMI.

### Further analysis of myelination in hypoxic mice treated with clemastine at the MED

**Fig. 3:**
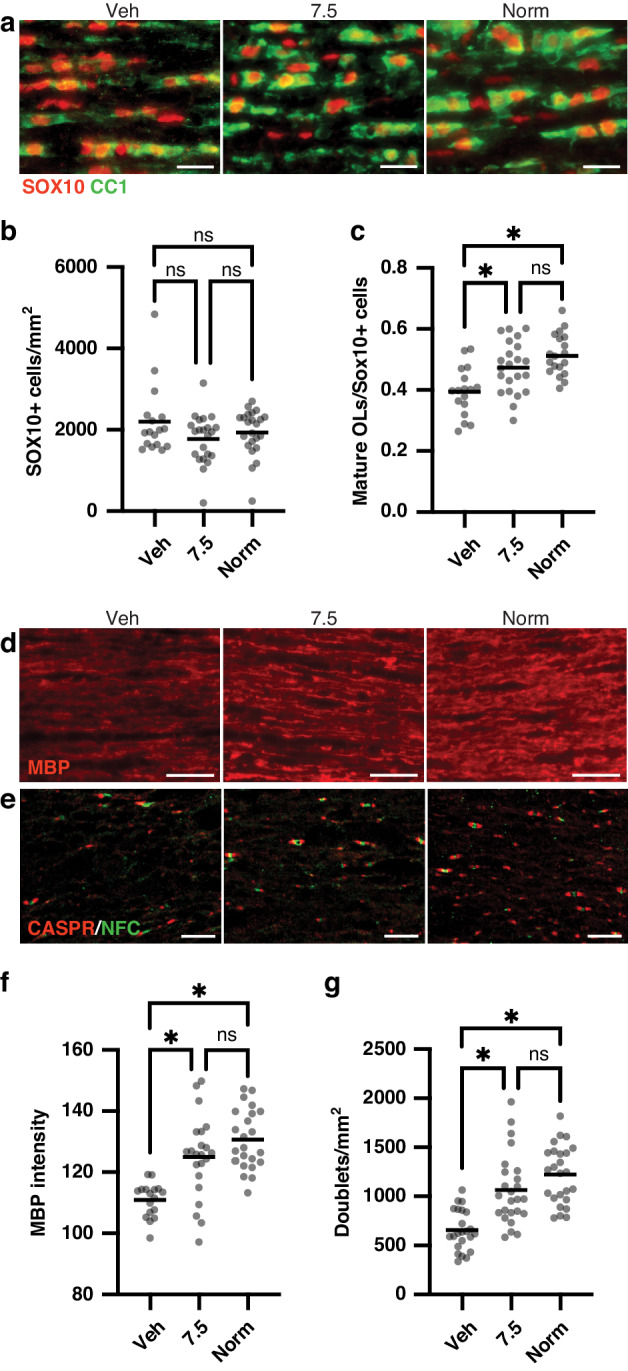
Clemastine treatment at the minimum effective dose rescues oligodendrocyte differentiation and myelination in the optic nerve at P14. **a** Representative images of staining for mature oligodendrocytes (CC1+ cells) and all oligodendrocyte lineage (SOX10+) cells in longitudinal sections of optic nerve at P14 in each treatment condition. Magnification: 20X; scale bars, 20 um. **b** Column scatter plot of SOX10+ cell density by treatment condition; data are compared by Kruskal–Wallis test. **c** Column scatter plot of proportion of mature (CC1+) oligodendrocytes out of all oligodendrocyte lineage (SOX10+) cells. *p < 0.05, one-way ANOVA followed by post hoc Tukey’s tests. **d** Representative images of staining for myelin basic protein (MBP) in P14 optic nerves. Magnification: 20X; scale bars, 30 um. **e** Representative images of staining for Caspr and neurofascin (NFC) to identify nodes of Ranvier in P14 optic nerves. Magnification: 40X; scale bars, 10 um. **f** Column scatter plot of normalized MBP intensity in P14 optic nerves. *p < 0.05, one-way ANOVA followed by post hoc Tukey’s tests. **g** Column scatter plot of density of nodes of Ranvier (identified by NFC/Caspr doublets) in P14 optic nerves. *p < 0.05, one-way ANOVA followed by post hoc Tukey’s tests. For all graphs, horizontal lines depict mean of data sets; ns not significant. For all panels, hypoxia-exposed animals were: Veh (vehicle-treated) and 7.5 (clemastine 7.5 mg/kg/day); Norm, normoxia-exposed animals.

We sought to confirm the promyelinating effects of clemastine at 7.5 mg/kg/day in the central nervous system. We assessed myelination and OL maturation in the optic nerves of hypoxia-exposed, vehicle-treated mice, normoxia-exposed (hereafter referred to as “normoxia”) mice, and hypoxia-exposed, clemastine-treated (“clemastine-MED”) mice (Fig. 3a–g and Supplementary Fig. 2a, b). Total OL lineage (SOX10+) cell density was not significantly different between groups (*p* = 0.34, Kruskal–Wallis test). Consistent with effects in the brain the proportion of mature OLs (CC1+ cells) out of all OL lineage cells and myelin protein expression (MBP intensity) were significantly higher in normoxia and clemastine-MED mice as compared to vehicle mice (*p* < 0.05, one-way ANOVAs followed by Tukey’s post hoc tests). The density of Nodes of Ranvier, as indicated by staining for Caspr and neurofascin, was also significantly higher in normoxia and clemastine-MED mice as compared to vehicle mice (*p* < 0.05, ANOVA followed by Tukey’s post hoc tests). OPC (PDGFRα+ cell) density was significantly lower in normoxia as compared to both vehicle and clemastine-MED mice (*p* < 0.05, Brown–Forsythe and Welch ANOVA followed by post hoc Dunnett’s test). Overall, these findings support the hypothesis that chronic hypoxia leads to an arrest of OL differentiation and a reduction in myelin formation, as previously reported,^7,8,12–14^ which are rescued by oral clemastine treatment at doses of 7.5 mg/kg/day and above.

**Fig. 4:**
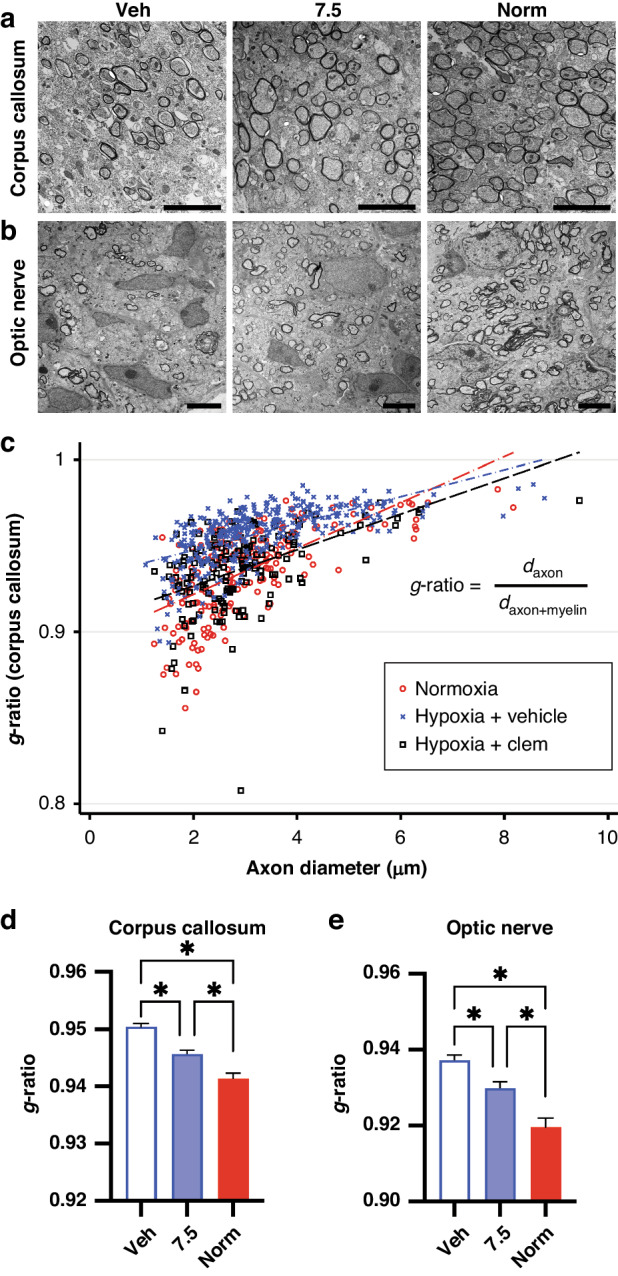
Clemastine improves myelin thickness after chronic hypoxia exposure in neonatal mice. **a** Representative electron microscopy images of the corpus callosum at 4700X magnification. Scale bars, 5 um. **b** Representative electron microscopy images of optic nerves at 2800X magnification. Scale bars, 5 um. **c** Scatter plot of g-ratios and axon diameters from the corpus callosum at P14, with superimposed linear regressions from normoxia-exposed (red), hypoxia-exposed/vehicle-treated (blue), and hypoxia-exposed/7.5 mg/kg/day clemastine-treated (black, “Hypoxia + Clem”) mice. d_axon_, diameter of axon; d_axon+myelin_, diameter of axon plus myelin sheath. **d** Bar plot comparing mean g-ratios in the corpus callosum. Error bars represent S.E.M. *p < 0.05, Kruskal–Wallis followed by post hoc Dunn’s tests. **e** Bar plot comparing mean g-ratios in optic nerves. Error bars represent S.E.M. *p < 0.05, Kruskal–Wallis followed by post hoc Dunn’s tests.

We asked whether clemastine treatment at the MED rescues hypoxia-induced changes in myelin ultrastructure.^21^ We measured myelin thickness at P14 in the corpus callosum and optic nerves by electron microscopy (EM) and performed g-ratio analysis, where the diameter of the axon is divided by the diameter of the axon plus the myelin sheath (Fig. 4a–e and Supplementary Fig. 2c). Clemastine treatment at the MED significantly improved (decreased) the mean g-ratio of axons in the corpus callosum and in optic nerves of hypoxia-exposed mice (*p* < 0.05, Kruskal–Wallis tests followed by Dunn’s post hoc tests). We also found that g-ratios in normoxia mice were significantly lower than g-ratios in clemastine-MED mice (*p* < 0.05, Kruskal–Wallis tests followed by Dunn’s post hoc tests). We concluded that clemastine treatment at the MED leads to a partial rescue of hypoxia-induced changes in myelin thickness at P14.

We asked whether hypoxia induces apoptosis in OL lineage cells, and whether hypoxia-induced apoptosis might be rescued by clemastine, an alternative hypothesis that could explain some of our observations. Apoptosis was assessed by staining for cleaved caspase-3 (CC3) in coronal brain sections at P14 (Supplementary Fig. 3a–c). No significant differences were observed across treatment conditions in the total density of CC3+ cells (*p* = 0.33, one-way ANOVA) or in the density of apoptotic OL lineage (OLIG2, CC3 double positive) cells (*p* = 0.34, one-way ANOVA). Importantly, these results do not eliminate the possibility that hypoxia induces OL lineage cell death at earlier ages or by caspase-independent mechanisms.^6^ However, in the context of prior in vitro and in vivo experimentation, direct induction of OL differentiation and myelination via M1 cholinergic receptor antagonism by clemastine is the most likely mechanism responsible for the rescue effects of clemastine observed in this study.^15,22^

### Clemastine has durable pro-myelinating effects in young adult mice after neonatal hypoxia

**Fig. 5:**
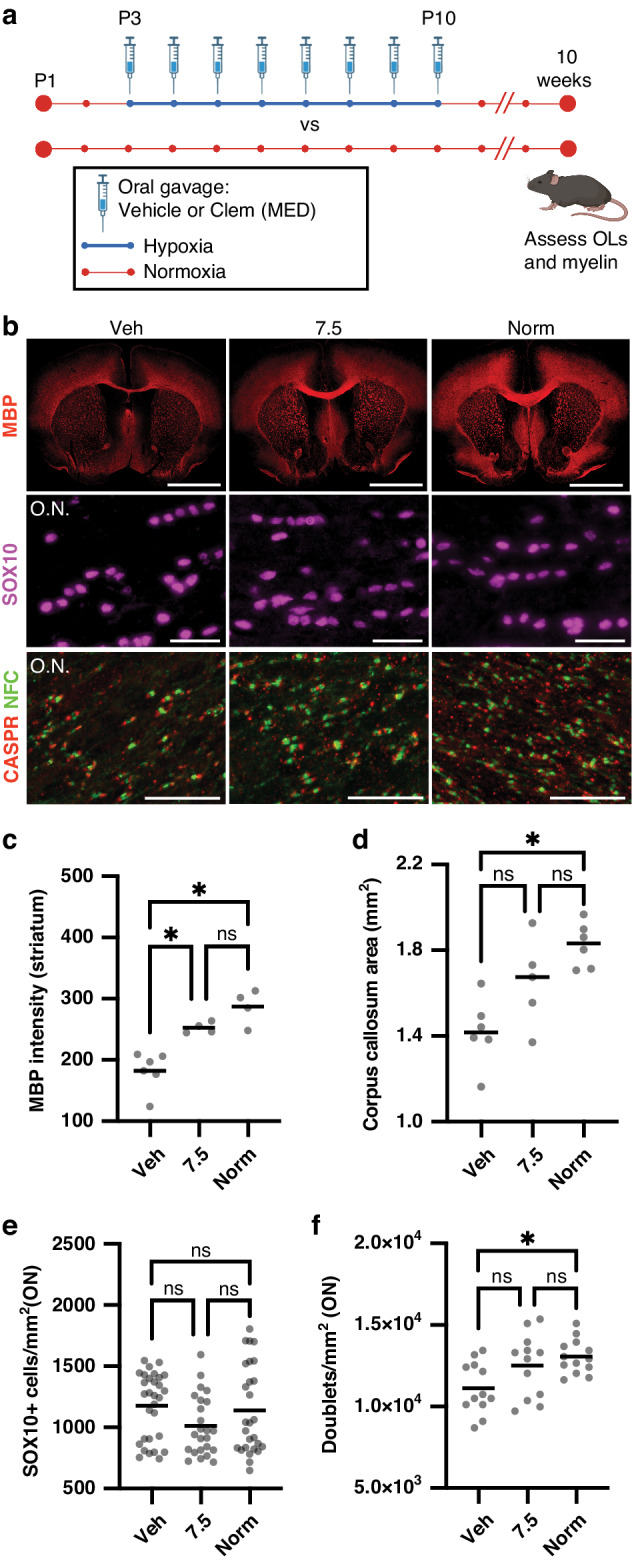
Clemastine treatment during neonatal hypoxia induces long-term improvements in myelination in young adult mice. **a** Experimental protocol for analysis of young adult mice. Mice were exposed to normoxia (21% FiO_2_) throughout the first two weeks of life, or to 10% FiO_2_ from postnatal day 3 (P3) through P10 while undergoing daily treatment with vehicle or clemastine (Clem) at 7.5 mg/kg/day. At 10 weeks of life, myelination and the oligodendrocyte (OL) lineage were assessed in all three treatment groups. MED, minimum effective dose. Created with BioRender.com. **b** Representative images of coronal brain sections stained for myelin basic protein (MBP, 10X magnification, scale bar: 1000 um), optic nerves stained for SOX10 (20X magnification, scale bar: 30 um) and optic nerves stained for Caspr and neurofascin (NFC) to identify nodes of Ranvier (40X magnification, scale bar: 30 um) in 10-week-old mice. O.N., optic nerve. **c** Column scatter plot of mean normalized MBP fluorescence intensity in the striatum in 10-week-old mice. *p < 0.05, one-way ANOVA followed by post hoc Tukey’s tests. **d** Column scatter plot of corpus callosum area quantified on MBP-stained coronal sections in 10-week-old mice. *p < 0.05, one-way ANOVA followed by post hoc Tukey’s tests. **e** Column scatter plot of SOX10+ cell density in optic nerves in 10-week-old mice. Median intensity was not significantly different between groups (*p* = 0.14, Kruskal–Wallis test). **f** Column scatter plot of density of nodes of Ranvier (identified by NFC/Caspr doublets) in P14 optic nerves. *p < 0.05, one-way ANOVA followed by post hoc Tukey’s tests. For all graphs, horizontal lines depict mean; ns, not significant. For all panels, hypoxia-exposed animals were: Veh (vehicle-treated) and 7.5 (clemastine 7.5 mg/kg/day); Norm, normoxia-exposed animals.

We assessed the long-term effects of clemastine treatment at the MED from P3-P10 on OL differentiation and myelination in young adult (10-week-old) mice after neonatal hypoxia exposure (Fig. 5a–f and Supplementary Fig. 4a–g). Myelin protein expression (MBP intensity) in the striatum was higher in normoxia and clemastine-MED mice as compared to vehicle mice (*p* < 0.05, one-way ANOVA followed by post hoc Tukey’s tests). MBP intensity in optic nerves was also higher in normoxia compared to vehicle mice (*p* < 0.05, one-way ANOVA followed by post hoc Tukey’s test), and was not significantly different between normoxia and clemastine-MED mice. Corpus callosum area was significantly greater in normoxia as compared to vehicle mice (*p* < 0.05, one-way ANOVA followed by post hoc Tukey’s test), and was not significantly different between normoxia and clemastine-MED mice. The density of Nodes of Ranvier, was also significantly higher in normoxia compared to vehicle mice (*p* < 0.05, ANOVA followed by Tukey’s post hoc tests), and was not significantly different between normoxia and clemastine-MED mice. The density of SOX10+ cells was not significantly different in the cortex (*p* = 0.25, Kruskal–Wallis test) or optic nerves (*p* = 0.14, Kruskal–Wallis test) across treatment conditions. Therefore, these findings could not be attributed to differences in total oligodendrocyte lineage cells. Axon density by neurofilament (NF) staining was also not significantly different in cortex (*p* = 0.14, Kruskal–Wallis test) or in optic nerves (*p* = 0.13, one-way ANOVA) across treatment conditions. Therefore, the promyelinating effects of clemastine are not secondary to differences in the number of axons available to wrap. Similar to results at P14, we found that OPC (PDGFRα+ cell) density in optic nerves was significantly higher in both clemastine-MED and vehicle mice as compared to normoxia mice (*p* < 0.05, Kruskal–Wallis test followed by post hoc Dunn’s tests). Thus, in some areas of the CNS, the pro-myelinating effects of clemastine may be attributable to increased myelin protein expression in OLs, or enhanced differentiation from premyelinating OLs to myelinating OLs.^36^ In summary, based on our analysis of 10-week-old mice, chronic hypoxia during the neonatal period results in sustained hypomyelination throughout the CNS, which is partially rescued by treatment with clemastine during the neonatal period.

### Pharmacokinetics of the minimum effective dose of clemastine

**Fig. 6:**
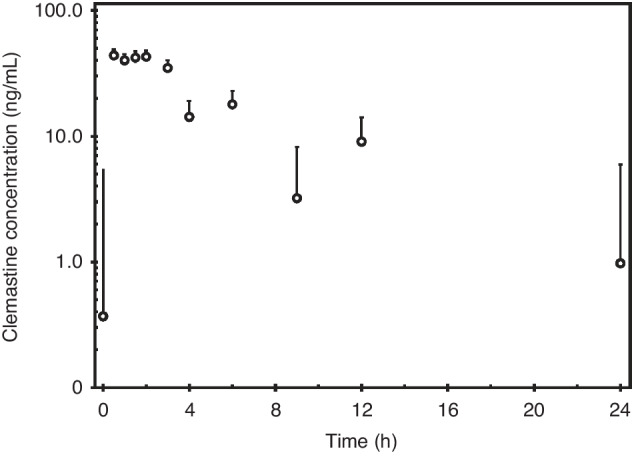
Plasma pharmacokinetics at steady-state of the minimum effective dose of oral clemastine in neonatal mice. Concentration-time curve of mean plasma clemastine concentration for mice treated with 7.5 mg/kg/day clemastine by oral gavage for 1 week. Error bars represent S.E.M.; *Y*-axis has a logarithmic scale. *N* = 4–5 mice per time point, with at least one mouse per sex included at each time point.

Neonatal drug development presents many challenges, as neonates often have altered absorption, distribution, metabolism, excretion, and toxicity profiles as compared to adults and older children.^37,38^ We sought to determine the pharmacokinetics of oral clemastine in neonatal mice. Fifty mice were treated daily with oral clemastine at the MED, 7.5 mg/kg, starting at P3. Mice were treated for one week to allow plasma concentrations to reach steady-state levels. Treated mice did not appear sedated and did not otherwise have any overt behavioral abnormalities. Mice were sacrificed at planned intervals before and up to 24 h following the 8th dose of clemastine for measurement of plasma clemastine concentrations by LC-MS/MS (Fig. 6). The plasma PK parameters are listed in Table 1. The area under the plasma concentration curve (AUC_24_) was 280.1 ng*hr / ml. The elimination half-life (t_1/2_) was 4.6 h, which is shorter than the t_1/2_ of 21.3 h previously published in adult humans and more similar to the elimination profile reported in dogs and horses.^39–41^ The short half-life resulted in low concentrations (0.7 to 1.4 ng/ml) at the end of the 24-h dosing interval and suggests that a shorter dosing interval (such as ≤ every 12 h) could further optimize the pro-myelinating effects of clemastine in rodent models of myelin disorders.

**Table 1:** Plasma pharmacokinetics of oral clemastine in neonatal mice.

Parameter	Value in Plasma	CV%
AUC_24_ (ng * h/mL)	280.1	*-*
C_max_ (ng/mL)	44.0	49.4%
T_max_ (hours)	0.5	*-*
C_24_ (ng/mL)	0.974	32.4%
T_1/2_ (hours)	4.6	*-*
Cl/F (L/h/kg)	26.8	*-*

Pharmacokinetic analysis was conducted using the geometric mean (geomean) of each timepoint. AUC_24_ = AUC_0-∞_ as plasma analysis was done at steady state. C_24_ refers to the trough concentration 24 h after oral administration. Cl/F (clearance/fraction) is the clearance of clemastine fumarate, as dosing was based on the weight of the fumarate salt. CV%, coefficient of variation; “-“, could not be calculated based on available data.

## Discussion

Current clinical management of PWMI is focused on treatment of symptoms such as spasticity and seizures, and early initiation of rehabilitative therapies. No medications are available that promote white matter repair or specifically target the oligodendroglial lineage. Translation of potential treatments from animal models to neonates has been impeded by ethical concerns, drug delivery challenges (such as unavailability or toxicity of liquid oral formulations or lack of blood-brain barrier penetration), and difficulty in predicting PK and toxicity profiles in neonates.^42–44^ Furthermore, identification of new therapies for PWMI and other myelin disorders has been slow due to technical barriers to high-throughput screening, such as a requirement for the presence of axons. Here, we demonstrate the MED and associated pharmacokinetics in neonatal mice of clemastine, an oral antihistamine with pro-myelinating properties identified using a novel high-throughput in vitro screening system.^15^ Clemastine strongly promotes myelination in multiple settings by directly targeting muscarinic receptors on OPCs.^17,18,20–22,32^ Previous mass spectrometry experiments demonstrate that clemastine efficiently penetrates the blood brain barrier and reaches the brain parenchyma.^45^ The medication has an excellent safety and toxicity profile when used for other indications, and is approved in children as young as 12 months old in many countries, with readily available liquid oral formulations. Clemastine may be positioned to overcome some of the challenges that have hindered translation of other potential therapies for PWMI.

We found that treatment with clemastine at the MED during the neonatal period rescued myelination and OL differentiation in P14 mice, an age where myelination is similar to that of a 1-year-old human.^27^ We also observed sustained deficits in myelination and OL differentiation in young adult animals after neonatal hypoxia exposure. These deficits were partially rescued by treatment with clemastine at the MED during the neonatal period. The literature contains variable reports regarding whether abnormalities in myelination and OL density persist in adult animals after neonatal hypoxia.^13,14,46,47^ The degree of spontaneous recovery of CNS myelination over time may depend on the specific anatomical region and age analyzed or on differences in experimental protocol. For example, our protocol does not include the use of foster mothers for pups exposed to hypoxia, a practice that appears to correlate with spontaneous recovery of myelination.^13,14^ Nevertheless, the durable therapeutic effect of clemastine observed in this study suggests that stimulating OL differentiation and myelination during the neonatal period has the potential to produce sustained, long-term repair in the setting of diffuse white matter injury, a finding with exciting therapeutic implications.

Several pharmacokinetic parameters identified in the current study should be considered in future clinical trial design. The CNS-penetrating, pro-myelinating effects of clemastine may require a minimum threshold total daily exposure (AUC_24_), maximum plasma concentration (C_max_), or trough plasma concentration (C_24_). Based on our results, the MED in neonatal mice provides a daily exposure that is approximately three times higher than the previously demonstrated myelination-promoting dose of clemastine in adult humans with multiple sclerosis in the ReBUILD trial.^39,48^ Three months of clemastine treatment were required to obtain evidence of remyelination in the ReBUILD trial.^48,49^ It is possible that higher daily clemastine doses in patients with multiple sclerosis would allow for a shorter treatment duration or enhanced remyelination. Alternatively, clemastine dosing requirements and ceiling effects may differ in multiple sclerosis as compared to PWMI due to distinct disease mechanisms, patient age, and inhibitory factors such as neuroinflammation. Additionally, while the MED identified in this study was 7.5 mg/kg/day, we could test only a limited number of doses due to issues of feasibility and space constraints in the hypoxia chamber. It is possible that the true MED lies between 2 and 7.5 mg/kg/day, a dose range that would encompass the exposure provided in the ReBUILD trial.

The current study has several additional limitations when considering translation to humans. We focused our study on assessment of myelination and OL maturation and did not examine behavioral outcomes in clemastine-treated mice. However, improvement in cognitive and motor outcomes after hypoxia has been previously demonstrated after one week of clemastine treatment at 10 mg/kg/day.^22^ Given the similarities in histological outcomes between the 7.5 and 10 mg/kg/day doses in our study, we would expect behavioral outcomes to be similar in both dose groups. Additionally, while we observed no overt signs of toxicity in mice treated with clemastine at any dose, we did not perform formal toxicity studies or determine the maximal tolerated dose in neonatal mice. Clemastine has side effects attributed to muscarinic antagonism, such as sedation, that may limit high doses in neonates. Future clinical trials in neonates must optimize daily exposure and plasma concentrations while limiting toxicity and the chance of adverse events. Safe clinical trial design will also require consideration of unique aspects of neonatal pharmacology; for example, medications metabolized in the liver display a slower rate of drug elimination in neonates as compared to older children, an observation attributed to liver immaturity.^50^ Overall, this study is a critical step towards development of a targeted treatment for PWMI. By identifying and characterizing the MED of clemastine that promotes recovery of hypomyelination in a murine chronic hypoxia model, we have overcome a significant barrier to initiation of a well-designed Phase I clinical trial in neonates with PWMI.

## Supplementary information

Supplementary_Table_1

Supplementary_Materials: Supplementary Figures 1 through 4 and Supplementary Tables 2 through 5

## Data Availability

The data generated during the current study are available from the corresponding author on reasonable request.
