# Supplementary material for: Minimum effective dose of clemastine in a mouse model of preterm white matter injury
Source: Pediatr Res. 2024 Jun 28;96(4):933–41. doi: 10.1038/s41390-024-03326-w (PMC11502491; doi:10.1038/s41390-024-03326-w)
Supplement: Supplementary file 2 — Supplementary_Materials: Supplementary Figures 1 through 4 and Supplementary Tables 2 through 5 [file 41390_2024_3326_MOESM2_ESM.pdf]

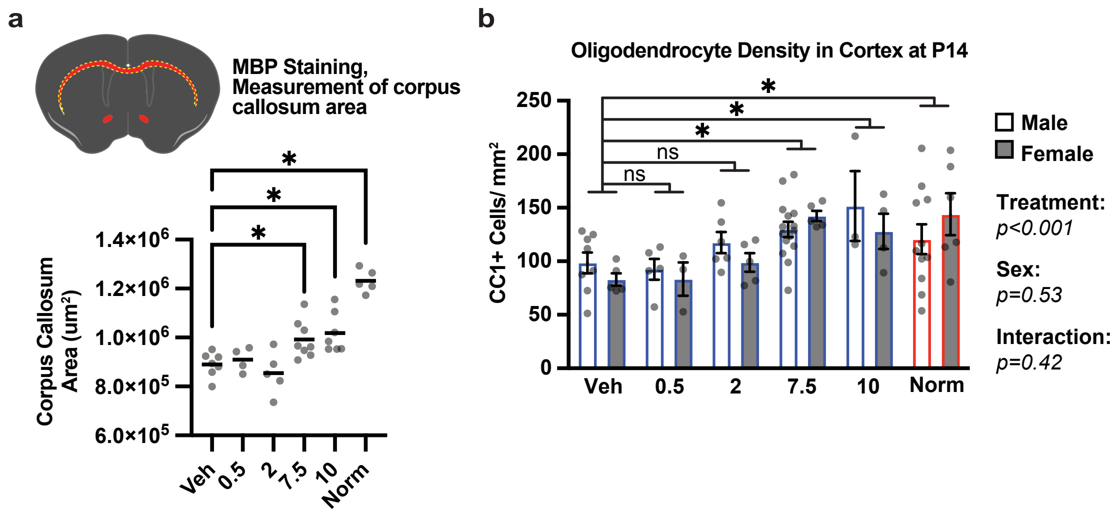

Supplementary Figure 1. Corpus callosum size and oligodendrocyte lineage analysis at P14 after chronic hypoxia. (a) Diagram of myelin basic protein (MBP) staining and corpus callosum area measurement, and column scatter plot of corpus callosum area in coronal brain sections at P14 after chronic hypoxia. Horizontal bars on graph indicate mean. *$p<0.05$, one-way ANOVA followed by post hoc Dunnett's tests. (b) Bar graph of mean mature oligodendrocyte (CC1+ cell) density in cortex by sex and treatment group. There was a significant effect of treatment group, but not sex or interaction, in a two-way ANOVA of treatment group and sex (predictors) with outcome of mature oligodendrocyte density. Results of pairwise comparisons between mean oligodendrocyte density of the vehicle group and other treatment groups are shown above the bar graph. *$p<0.05$, two-way ANOVA followed by post hoc Dunnett's tests; ns, not significant. Error bars represent standard error of the mean. For all graphs, hypoxia-exposed animals were: Veh (vehicle-treated), 0.5 (clemastine 0.5 mg/kg/day), 2 (clemastine 2 mg/kg/day), 7.5 (clemastine 7.5 mg/kg/day), 10 (clemastine 10 mg/kg/day). Norm, normoxia-exposed animals.

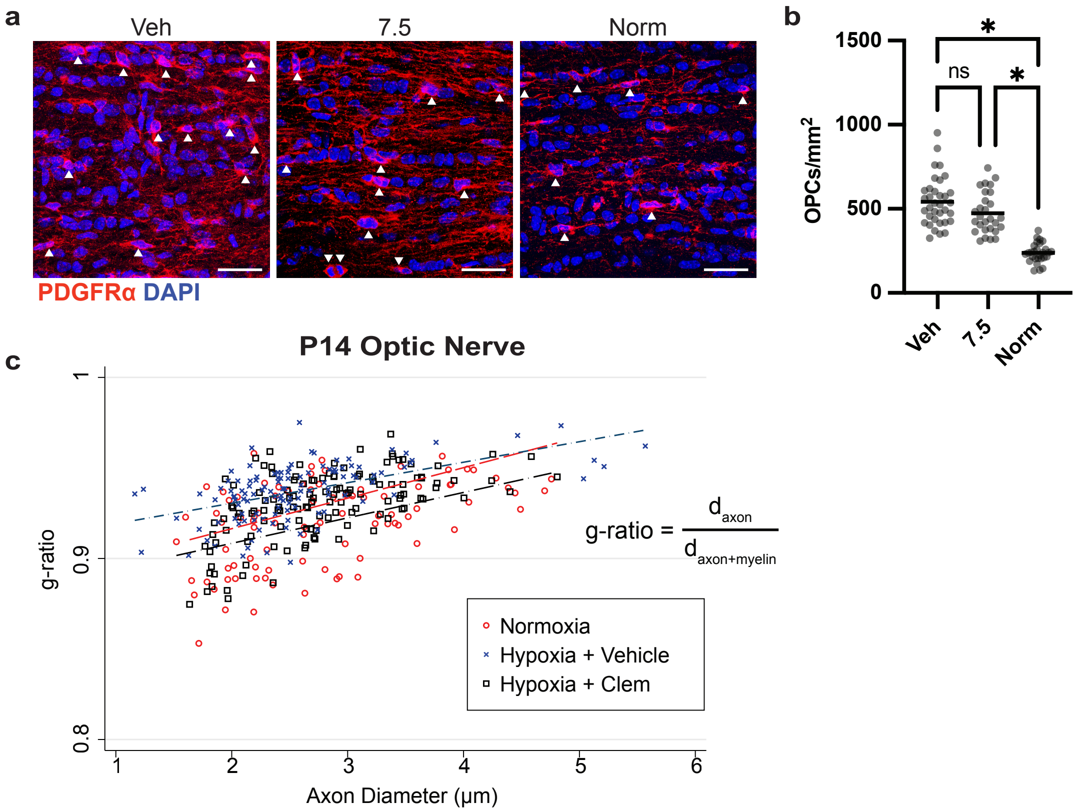

Supplementary Figure 2. Additional analysis of P14 optic nerves after chronic hypoxia. (a) Representative images of oligodendrocyte precursor cell (OPC) density by PDGFRα+ staining. Arrowheads, OPCs; magnification, 40X; Scale bars, 20um. (b) Column scatter plot of OPC (PDGFRα+ cell) density in optic nerves at P14. Horizontal lines indicate mean. *$p<0.05$, Brown-Forsythe and Welch ANOVA followed by post hoc Dunnett's tests. For *a* and *b*, hypoxia-exposed animals were: Veh (vehicle-treated) and 7.5 (clemastine 7.5 mg/kg/day); Norm, normoxia-exposed animals. (c) Scatter plot of g-ratios and axon diameters from the optic nerve at P14, with superimposed linear regressions from normoxia-exposed (red), hypoxia-exposed/vehicle-treated (blue), and hypoxia-exposed/7.5 mg/kg/day clemastine-treated (black, "Hypoxia + Clem") mice. d_{axon}, diameter of axon; $d_{axon+myelin}$, diameter of axon plus myelin sheath.

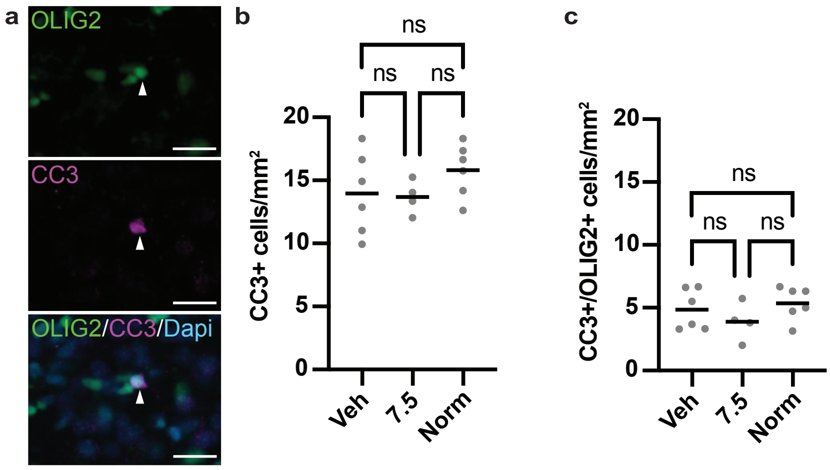

Supplementary Figure 3. Analysis of apoptosis after hypoxia and clemastine treatment. (a) Example image of cleaved caspase-3 (CC3)-positive oligodendrocyte lineage (OLIG2+) cell in the periventricular region of a P14 hypoxia-exposed mouse treated with 7.5 mg/kg/day clemastine. Magnification: 20X; scale bars, 50 um. Apoptotic cells were quantified in one coronal brain section per mouse as CC3+ cells per area (b) and CC3+ oligodendrocyte lineage (CC3, OLIG2 double positive) cells per area (c) in Veh (hypoxia-exposed, vehicle-treated), 7.5 (hypoxia-exposed, 7.5mg/kg/day clemastine-treated) and Norm (normoxia-exposed) mice. Horizontal bars show mean of each treatment group. For both (b) and (c), no significant differences were observed between conditions using one-way ANOVA followed by Tukey's post hoc tests (ns, not significant).

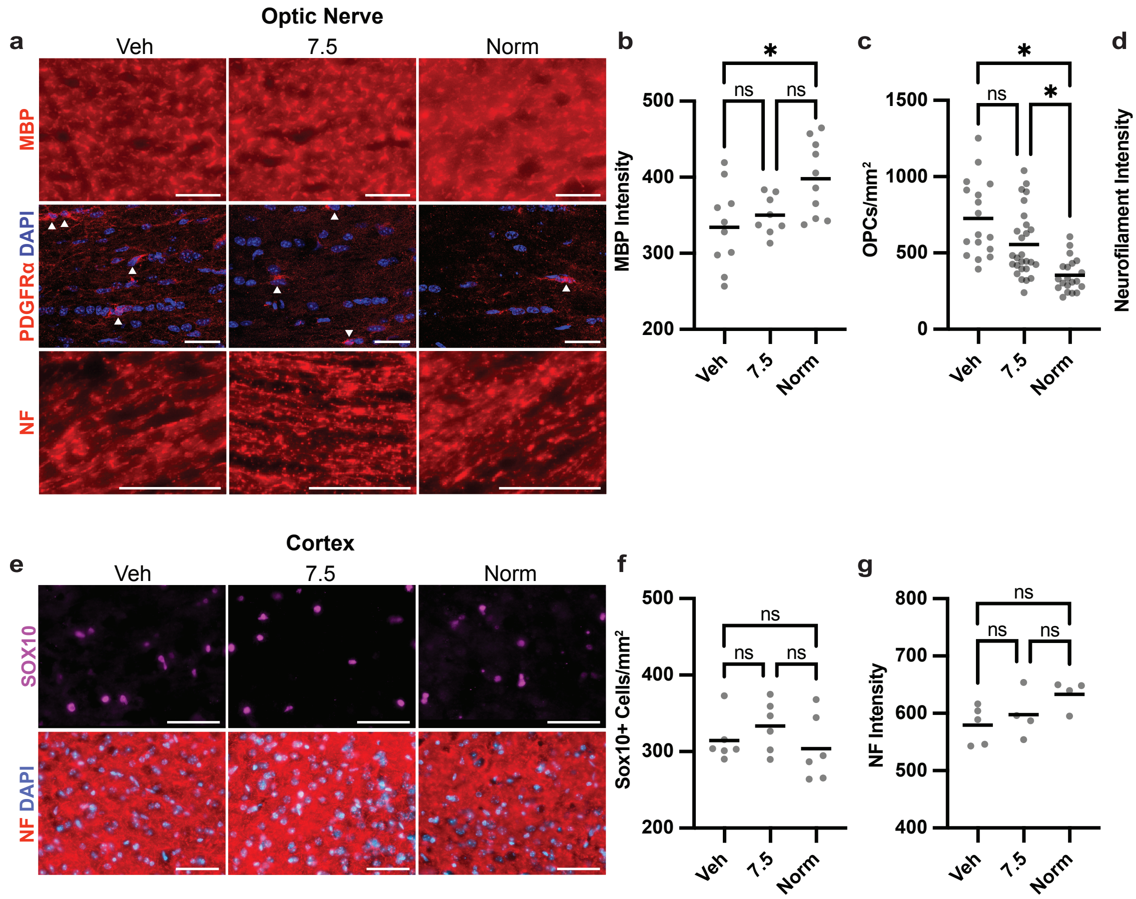

Supplementary Figure 4. Additional analysis of myelination and the oligodendrocyte lineage in young adult mice after clemastine treatment during neonatal hypoxia. (a) Representative images of staining for myelin basic protein (MBP, 20X magnification), OPCs (PDGFRα+ cells, 40X magnification), and neurofilament (NF, 20X magnification) in optic nerves in 10 week old mice. Scale bars, 30 um. (b) Column scatter plot of normalized MBP intensity in optic nerves of 10 week old mice. *$p<0.05$, one-way ANOVA followed by post hoc Tukey's tests. (c) Column scatter plot of OPC (PDGFRα+ cell) density in optic nerves in 10 week old mice. *$p<0.05$, Kruskal-Wallis test followed by post hoc Dunn's tests. (d) Column scatter plot of normalized neurofilament intensity by treatment group. Median intensity was not significantly different between groups ($p=0.14$, Kruskal-Wallis test). (e) Representative images of staining for oligodendrocyte lineage (SOX10+) cells and neurofilament (NF) in cortex in 10 week old mice. Magnification: 20X; scale bars, 50 um. (f) Column scatter plot of SOX10+ cell density by treatment group. Median intensity was not significantly different between groups ($p=0.28$, Kruskal-Wallis test). (g) Column scatter plot of normalized neurofilament intensity by treatment group. Median intensity was not significantly different between groups ($p=0.20$, Kruskal-Wallis test). For all column scatter plots, horizontal lines depict mean; ns, not significant. For all panels, hypoxia-exposed animals were: Veh (vehicle-treated) and 7.5 (clemastine 7.5 mg/kg/day); Norm, normoxia-exposed animals.

Supplementary Table 2. Two-way ANOVA results, analysis of the effect of treatment group and sex on myelination (MBP intensity in striatum).

Source	SS	df	MS	F-value	p-value
**Sex**	1783	1	1783	0.650	0.424
**Treatment Group**	391150	5	78230	28.51	<0.001
**Interaction**	10168	5	2034	0.741	0.596
**Residual**	150927	55	2744		

Note. SS, sum of squares (Type III); df, degrees of freedom; MS, mean square.

Supplementary Table 3. Two-way ANOVA results, analysis of the effect of treatment group and sex on mature (CC1+) oligodendrocyte cell density in cortex.

Source	SS	df	MS	F-value	p-value
**Sex**	0.006	1	0.006	0.396	0.531
**Treatment Group**	0.414	5	0.083	5.441	<0.001
**Interaction**	0.076	5	0.015	0.999	0.425
**Residual**	0.974	64	0.015		

Note. Two-way ANOVA performed on log transformed data which passed Shapiro-Wilk tests for normality of the residuals, and Spearman's test for heteroscedasticity. SS, sum of squares (Type III); df, degrees of freedom; MS, mean square.

Supplementary Table 4. Two-way ANOVA results, analysis of the effect of treatment group and sex on total SOX10+ oligodendrocyte lineage cell density in cortex.

Source	SS	df	MS	F-value	p-value
**Sex**	3876	1	3876	0.917	0.342
**Treatment Group**	44515	5	8903	2.106	0.076
**Interaction**	18009	5	3602	0.852	0.518
**Residual**	270589	64	4228		

Note. SS, sum of squares (Type III); df, degrees of freedom; MS, mean square.

Supplementary Table 5. Two-way ANOVA results, analysis of the effect of treatment group and sex on proportion of oligodendrocye precursor cells (CC1-/SOX10+ cells) out of all oligodendrocyte lineage cells (SOX10+ cells) in cortex.

Source	SS	df	MS	F-value	p-value
**Sex**	0.006	1	0.006	0.747	0.390
**Treatment Group**	0.414	5	0.083	10.98	<0.001
**Interaction**	0.041	5	0.008	1.079	0.380
**Residual**	0.482	64	0.007		

Note. SS, sum of squares (Type III); df, degrees of freedom; MS, mean square.
